# High-frequency plasma exchange therapy for immunocompromised, type I crescentic glomerulonephritis complicated with IgA nephropathy: A case report and literature review

**DOI:** 10.1097/MD.0000000000032698

**Published:** 2023-01-20

**Authors:** Huihui Chen, Jingjing Jin, Mei Juan Cheng, Lei He, Wei Zhou, Liping Guo, Zhe Zhe Niu, Xiang Nan Liang, Rong Fang Zhu, Yaling Bai, Jin Sheng Xu

**Affiliations:** a Hebei Clinical Research Center for Chronic Kidney Disease, Hebei Key Laboratory of Vascular Calcification in Kidney Disease, Department of Nephrology, The Fourth Hospital of Hebei Medical University, Shijiazhuang, P.R. China.

**Keywords:** anti-glomerular basement membrane disease, case report, IgA nephropathy, plasma exchange, type I crescentic glomerulonephritis

## Abstract

Anti-glomerular basement membrane (anti-GBM) disease has been reported to coexist with other immune-mediated glomerular disorders, including antineutrophil cytoplasmic autoantibody positive glomerulonephritis and membranous glomerulopathy. It is well known that anti-GBM disease often manifests as type I crescentic glomerulonephritis on renal biopsy. However, concurrent cases of both type I crescentic glomerulonephritis and IgA nephropathy are rare.

**Patient concerns:**

We report the case of a 40-years-old woman with microscopic hematuria, mild proteinuria and an immunocompromised status. Laboratory data revealed serum creatinine showed progressive progress, suddenly rising from the normal range to 316.2*μ*mol/L within 4 months. The CD4 lymphocyte count was 0.274 × 10^9^/L (reference value 0.35–1.82 × 10^9^/L). The anti-GBM antibody titer was 192.4 IU/mL (reference range: <20 RU/mL).

**Diagnoses:**

Renal biopsy was performed after admission. The pathological diagnosis was type I crescentic glomerulonephritis, IgA nephropathy, and clinical anti-GBM disease.

**Interventions:**

The patient was seriously ill on admission and progressed rapidly. Combined with poor immune function, we immediately initiated high-frequency plasma exchange (PE). In addition, to avoid rebound of antibody levels, PE was performed for 5 times. Follow-up treatment was combined with standard-dose corticosteroids and cyclophosphamide.

**Outcomes:**

The patient was followed up for 1 year. On the last visit, her serum creatinine decreased to 103.5*μ*mol/L, anti-GBM antibody remained negative, and proteinuria and hematuria disappeared.

**Lessons:**

This case illustrates that when crescentic nephritis or anti-GBM disease is combined with other immune diseases, especially when the immune function is extremely low, if the application of high-dose steroid shocks may induce fatal infections, to some extent high frequency PE has certain advantages.

## 1. Introduction

Crescentic glomerulonephritis also known as rapidly progressive glomerulonephritis, is characterized by the presence of numerous glomerular crescents. Type I crescentic glomerulonephritis is one of the 5 immunopathological types of crescentic glomerulonephritis. It is usually characterized by linear deposits of immunoglobulin G (IgG) along the capillary loop, and the serological detection of anti-glomerular basement membrane (anti-GBM) antibody, so it is also called anti-GBM disease.^[1]^ McAdoo SP et al^[2]^ showed that the non-collagen (NC1) domain of the *α*3 chain of type IV collagen is the main target of autoimmune responses against anti-GBM disease. Anti-GBM disease has been reported to coexist with pauci-immune antineutrophil cytoplasmic autoantibody-positive glomerulonephritis and membranous glomerulopathy. Here we describe an unusual case of renal disease in which antibodies against glomerular basement membrane (GBM) were detected in serum with histological evidence of type I crescentic glomerulonephritis and IgA nephropathy, and poor immune function. In the following case, we first applied high-frequency plasma exchange (PE) combined with corticosteroids and cyclophosphamide, and the patient’s renal function had gradually returned to near normal.

## 2. Case presentation

A 40-years-old Chinese female was admitted to our hospital on April 30, 2021 with fatigue, increasing foam in the urine that had been lasting for 4 months. For 4 months before admission, the patient experienced fatigue, increasing foam in the urine and the symptoms could not be alleviated. She did not cough or show any symptoms of dyspnea or hemoptysis. She previously underwent ovariectomies at a local hospital 12 years ago and cesarean section 16 years ago. Allergic to penicillin and cefixime. There was no history of recent flu-like illness or other infection. She had no other occupational exposures relevant to these respiratory symptoms. There was no other significant medical history, including no history of previously detected hematuria or proteinuria. Physical examination showed no significant abnormalities (there were no peripheral edema). His blood pressure was 121/80 mm Hg, her pulse was 84 beats per minute, his peripheral oxygen saturation was 96% in ambient air, and his temperature was 37.1°C. Laboratory analysis revealed serum creatinine showed progressive progress, suddenly rising from the normal range to 316.2*μ*mol/L within 4 months. Urinalysis showed 1700 red blood cells per *μ*l with mild proteinuria (0.941 g per day). ESR was raised at 112.5 mm/hours, a serum albumin of 34.8 g/L; On admission, the CD4 lymphocyte count was 0.274 × 10^9^/L (reference value 0.35–1.82 × 10^9^/L). In addition, ANA and dsDNA antibodies were not elevated. Both an ENA screen and antineutrophil cytoplasmic antibody (ANCA) antibodies were also negative. Other parameters are shown in (Table 1). Lung computed tomography showed there were multiple ground glass nodules in the lower lobe of the right lung. The length and diameter of the nodules were about 3 to 4 mm, which were small and considered benign. Annual review was established. Further diagnostic work-up revealed the presence of anti-GBM antibody at the titer of 192.4 IU/mL (reference range: <20 RU/mL). ANA and dsDNA antibodies were not elevated. Both an ENA screen and ANCA antibodies were also negative.

**Table 1:** Laboratory data on admission.

Variable	Day of admission	Reference range
CBC		
WBC (*10^9^/L)	10.63	3.5–9.5
Hgb (g/L)	99	115–150
PLT (*10^9^/L)	395	125–350
Biochemical		
ALB (g/L)	34.8	40–55
Creatinine (μmol/L)	262.7	41–73
BUN (mmol/L)	9.7	2.6–7.5
Na (mmol/L)	140	137–147
K (mmol/L)	4.3	3.5–5.3
Ca (mmol/L)	2.29	2.11–2.52
P (mmol/L)	1.59	0.85–1.51
Immune-related		
IgG (g/L)	11.2	8.6–17.4
IgA (g/L)	3.83	1.0–4.2
IgM (g/L)	0.89	0.5–2.8
C3 (g/L)	1.41	0.7–1.4
C4 (g/L)	0.36	0.1–0.4
Anti-GBM antibody titer (RU/ml)	192.4	<20
PR3-ANCA (RU/mL)	<2	<20
MPO-ANCA (RU/mL)	<2	<20
ESR (mm/h)	112.5	0–20
CRP (mg/L)	86.7	0–6
Urinalysis		
Occult blood	3+	
RBCs (/μL)	1701.48	0–10
Protein	1+	
24 h urine protein/ creatinine (mg/g)	1045	≤200
WBC	56.43	0–12

ALB = albumin, ANCA = antineutrophil cytoplasmic antibody, BUN = blood urea nitrogen, Ca = calcium, CRP = C-reactive protein, GBM = glomerular basement membrane, Hgb = hemoglobin, IgG = immunoglobulin G, K = potassium, MPO = myeloperoxidase, Na = sodium, P = phosphate, PR3 = proteinase3, PLT = platelets, WBC = white blood cells.

**Figure 1.**
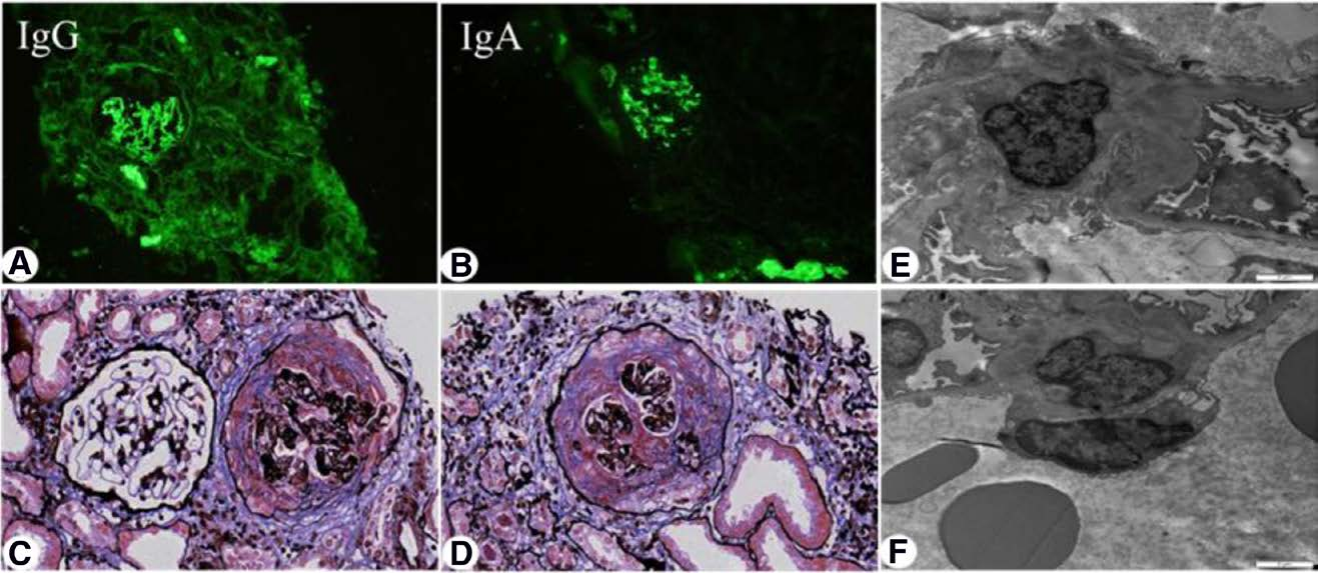
Renal biopsy findings. (a). Direct immunofluorescence analysis showed strong (+++) linear staining along the capillary loop for IgG (×200). (b) Direct immunofluorescence analysis showed strong (+++) granular staining along the mesangium for IgA (×200). (c–d) Light microscopy of renal puncture tissue revealed the formation of large crescents, the pathological manifestations were dominated by cellular crescents (×400). (e–f) Electron microscopic photograph of the renal biopsy, showing the electron-dense deposits in mesangial areas (×6000). IgG = immunoglobulin G.

The renal biopsy performed on the 11th day after admission. Light microscopy revealed crescents in 27 of 35 glomeruli(Fig.1c–d), of which 2 were glomerular sclerosis, and the rest of the glomerular capillary loops were diffusely severely damaged, compressed, and 2 glomeruli were segmental fibrinoid Necrosis, eosinophilic deposition in mesangial area; 2 cellular, 14 cellular fibrous, 8 fibrous, 1 microcellular, 2 microcellular fibrous crescents formation, empty tubular epithelial cells Degeneration of vesicles and granules, multifocal and patchy atrophy, and multifocal and patchy lymphoid and mononuclear cell infiltration in the renal interstitial with fibrosis. The walls of the arterioles were thickened and the lumen narrowed. Immunofluorescence demonstrated linear staining with IgG (Fig.1a–b) along the capillary loop and as well as prominent mesangial staining with IgA and C3. Electron microscope (Fig.1e–f) showed the proliferation of mesangial cells and matrix, the deposition of electron dense material in the mesangial, the segmental fusion of epithelial cell foot processes, and no special lesions in renal tubules and interstitial. The pathological diagnosis was type I crescentic glomerulonephritis, IgA nephropathy, and clinical anti-GBM disease. The final diagnosis was Type I crescentic glomerulonephritis complicated with IgA nephropathy.

**Figure 2.**
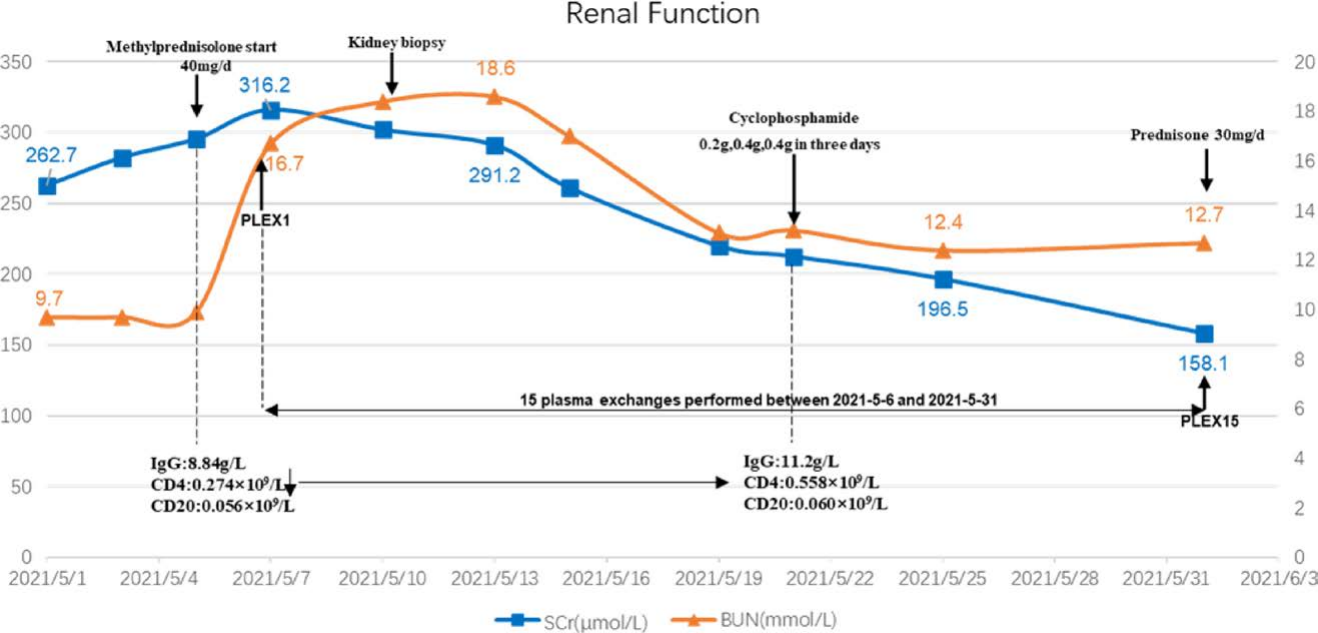
The patient’s renal function during hospitalization. Serum creatinine (*μ*mol/L) and blood urea nitrogen (mmol/L) by hospital day. BUN = blood urea nitrogen, CD4 = T4 lymphocyte counts, CD20 = B lymphocytic counts, IgG = immunoglobulin G, PLEX = plasma exchange, SCr = serum creatinine.

Given that the patient was seriously ill at admission and progressed rapidly, manifesting as rapidly progressive glomerulonephritis, renal biopsy showed that the proportion of crescents was high, reaching 77.1%. Combined with poor immune function of the patient, the CD4 lymphocyte count was 0.274 × 10^9^/L, which was significantly lower than normal, so we did not give methylprednisolone pulse therapy. We immediately initiated high-frequency PE (single plasmapheresis, exchange dosage: 2000 mL fresh frozen plasma), and the anti-GBM antibody levels were fully suppressed after 10 days. In addition, to avoid rebound of antibody levels, PE was performed 5 times. Meanwhile, methylprednisolone for intravenous infusion was commenced at 40 mg daily, tapering to oral prednisolone 30 mg daily at 1 months and tapering to 10 mg daily at 3 months and with intention to cease at 7 months. After 14 days of given methylprednisolone 40 mg/d, the patient’s CD4 cell count increased from 0.274 × 10^9^/L to 0.558 × 10^9^/L, and then we gave intravenous cyclophosphamide treatment. In order to ensure the patient’s safety and treatment effect, cyclophosphamide 0.2 g, 0.4 g, 0.4 g was administered in 3 days. Thereafter, intravenous infusion of cyclophosphamide 800 mg monthly. Up to now, the cumulative dose of cyclophosphamide has reached 5800 mg. During PE, the patient’s serum creatinine decreased from 316.2 *μ*mol/L to 158.1 *μ*mol/L, and antibody titer of GBM decreased from 192.4 IU/mL to 2.5 IU/mL. After that, no blood purification treatment was performed. After stopping PE, serum creatinine gradually decreased to 137.5 *μ*mol/L in 3 months, and antibody titer of GBM remained negative, urine protein gradually decreased to 148 mg/24 hour. The patient was followed up for 1 year. On the last visit, her serum creatinine decreased to 103.5 *μ*mol/L, anti-GBM antibody remained negative, and proteinuria and hematuria disappeared. Fig.2 summarizes the patient’s clinical course.

## 3. Discussion

Some studies have reported anti-GBM disease can be complicated by other immune-induced glomerular diseases. ANCA-associated vasculitis and membranous nephropathy are the most common^.[1,2]^ However, cases of anti-GBM disease coexistence with IgA nephropathy are rare. In our case, the patient’s renal immunopathology showed diffuse mass deposition of IgA along the mesangial area, IgG was linearly deposited along the glomerular capillary loop; light microscopy pathology was consistent with crescentic nephritis, and circulating anti-GBM antibodies with high titers were found. Therefore, we diagnosed the patient with Type I crescentic glomerulonephritis and IgA nephropathy. At present, there is no clear guideline or consensus on the treatment of type I crescentic glomerulonephritis and IgA nephropathy. Combined with literature review^[3–13]^ (Table 2), most patients present with extensive glomerular crescent formation, clinically characterized by rapidly progressive GN, due to the acute onset of anti-GBM disease and the severe condition, the treatment should be carried out according to the anti-GBM disease treatment plan first. Standard treatment for anti-GBM disease includes plasmapheresis therapy, which aims to rapidly remove pathogenic autoantibodies; Cyclophosphamide and corticosteroids are also included to inhibit further autoantibody production and improve end-organ inflammation. An observational study by Levy JB et al^[14]^ supported plasma replacement therapy, which showed improved kidney and patient survival compared to a historical cohort receiving immunosuppressive therapy alone. Moreover, a large Chinese study of 221 patients by Cui Z et al^[15]^ suggested that patients receiving plasma replacement had a better prognosis than those treated with cytotoxicity and corticosteroids alone.

**Table 2:** Literature review on clinical features and treatment prognosis of type I crescentic glomerulonephritis complicated with IgA nephropathy.

Case	Gender	Age	Admission highest creatinine(*μ*mol/L)	Anti-GBM antibody (RU/mL)	Treatment	Outcome
1^4^	Male	41	720	+	Plasmapheresis, Cyclophosphamide steroids	Loss to follow- up
3^5^	Male	27	1347	+,134%	/	Maintenance dialysis (MHD)
4^6^	Female	38	505	237.6	Methylprednisolone 750 mg/d × 5d CTX 0.2 g every other d	Anti-GBM antibody rechecked negative after 4 mo, creatinine was 196*μ*mol/L.
5^7^	Female	31	287	93.5	Methylprednisolone 0.5g*3 courses plasma exchange 10 times; Oral CTX 1 mg/kg/d prednisone 1mg/kg/d intravenous	Anti-GBM antibody 19.7RU/mL after 29 d, creatinine was 375*μ*mol/L.
7^8^	Male	24	1387.88	+	Pulse dose of intravenous methylprednisolone 500 mg/d for three days followed by maintenance oral prednisolone at 1 mg/kg/day and intravenous cyclophosphamide (10 mg/kg) every two wk	MHD
8^9^	Female	50	232	258.3	Pulse dose of intravenous methylprednisolone/sequential steroid combined with MMF	Creatinine rechecked was 74 mmol/L after 2 yr.
9^12^	Female	22	282.88	96	Three pulses of methylprednisolone 500 mg/d followed by oral prednisolone 0.5 mg/kg/d	Renal failure
11^2^	Female	66	488.85	116	PE/ Blood purification	MHD
12^11^	Male	22	77	+	PE/sequential steroid combined with cyclophosphamide/sequential steroid combined with azathioprine 2 mo later	The patient was admitted with respiratory symptoms and renal condition was basically stable.
13^10^	Female	38	481.78	187.2	Pulse dose of intravenous methylprednisolone and cyclophosphamide	Anti-GBM antibody turned negative after 3 mo, creatinine was 183.88 *μ*mol/L.
15^3^	Female	41	374	77.107	Pulse dose of intravenous methylprednisolone/rituximab (RTX)/PE/ blood purification/IVIG/ sequential steroid combined with tacrolimus (TAC)	Creatinine rechecked was 151.7 *μ*mol/L after 28 wk with hematuria and proteinuria improved significantly.
16 (this case)	Female	40	316.2	192.4	Intravenous methylprednisolone/PE/sequential steroid combined with cyclophosphamide	Anti-GBM antibody turned negative; the patient’s serum creatinine dropped to 109.7 *μ*mol/L after 1yr.

GBM = glomerular basement membrane, PE = plasma exchange.

Standard treatment regimens require oral prednisolone 1 mg/kg daily (maximum 60 mg). The dosage was reduced to 20 mg at 6 weeks, then gradually reduced until complete cessation at 6 to 9 month. Zhang et al^[4]^ recently reported pneumocystis pneumonia occurred following intensive immunosuppressive therapy for anti-GBM disease with IgA nephropathy. In the above medical record, the patient was treated with pulsed dose intravenous methylprednisolone, immunosuppression combined with rituximab, and plasmapheresis. This study also suggested severe immunosuppression and a high risk of secondary pneumocystis opportunistic infection. In contrast, in our case, the patient was seriously ill, and immunocompromised on admission, CD4 lymphocyte count was 0.274 × 10^9^/L, which was significantly lower than the normal value. Combined with the pathological results of renal biopsy showed that the proportion of crescents was high, reaching 77.1%, so pulse methylprednisolone was not given. We started with a standard dose of methylprednisolone 40 mg/day combined with high-frequency PE, after active treatment in the early stage for 14 days. When the immune function of the patient recovered, then we combined with cyclophosphamide treatment. During the whole treatment process, the patient did not develop symptoms such as infection.

In conclusion, through this case, we suggest that the patient’s immune function is poor, and the risk of methylprednisolone pulse therapy is high. Therefore, the patient in this case was not treated with methylprednisolone pulse therapy. Considering the relevant indicators of the patient, we will think about increasing the frequency of PE and not using methylprednisolone pulse therapy. Maybe in the future similar cases have the same benefit in treatment, and at the same time, fatal infections (such as Pneumocystis carinii pneumonia) caused by pulse glucocorticoid therapy can be avoided. Moreover, we still need more prospective studies to prove our point.

## Author contributions

**Project administration:** Jin sheng Xu.

**Supervision:** Jin sheng Xu.

**Writing – original draft:** Huihui Chen.

**Writing – review & editing:** Yaling Bai, Jingjing Jin, Mei juan Cheng, Lei He, Wei Zhou, Liping Guo, Zhe zhe Niu, Xiang nan Liang, Rong fang Zhu.
